# Exploring the origin and conceptual framework of the EQ VAS

**DOI:** 10.1007/s11136-025-03947-6

**Published:** 2025-04-26

**Authors:** Ling-Hsiang Chuang, Paul Kind, Thomas Kohlmann, You-Shan Feng

**Affiliations:** 1 Department of Epidemiology and Global Health, Umeå University, Umea, Sweden; 2 LHC Healthcare Consultancy, IJsselstein, Netherlands; 3 Academic Unit of Health Economics, University of Leeds, Leeds, UK; 4 Department of Applied Health Research, UCL, London, UK; 5 Institute for Community Medicine, Universitätsmedizin Greifswald, Greifswald, Germany; 6 Institute for Clinical Epidemiology and Applied Biostatistics, Universitätsklinikum Tübingen, Tübingen, Germany

**Keywords:** EQ VAS, Visual analogue scale, VAS, EQ-5D, Framework

## Abstract

**Purpose:**

The objective of this paper is to report on the origin of the EQ VAS and current understanding of the EQ VAS conceptual framework via a literature search based on the snowball approach.

**Methods:**

A review was conducted in two steps: (1) a citation search and (2) a search of the EuroQol group’s grey literature.

**Results:**

The findings indicate that the EQ VAS was originally designed as a warm-up task for valuing hypothetical health states. The characters of the EQ VAS reflect its valuation origin, such as drawing a line (the previous version), vertical orientation, and choice of end labels. None of these design elements of the EQ VAS were chosen for the purpose of measuring self-reported overall health. Despite this, the EQ VAS proves to be a valid self-reported health status measure with its psychometric properties demonstrated in various general and clinical populations. We found a dearth of literature addressing the conceptual framework of EQ VAS as a measure of self-rated overall health.

**Conclusion:**

With its potential as a powerful measure of overall health, further research into EQ VAS design, conceptual framework and empirical function is warranted.

**Supplementary Information:**

The online version contains supplementary material available at 10.1007/s11136-025-03947-6.

## Background

Visual analogue scales (VAS) are a class of measurement tools used to record subjective assessments of phenomena related to personal experience across diverse settings including consumer satisfaction, political and social surveys, and symptom severity. VAS can also be referred to as “category rating scales” or “rating scales”. Although lacking a uniform definition, a VAS is usually presented as a line, which may vary in length, with endpoints labelled to describe the boundaries of the phenomena of interest. Intermediate locations along the scale can also be labelled. For example, the “graphic rating scale” [1], one of the earliest forms of VAS, is a line with text descriptors along its length. Participants self-report using the VAS by placing a mark on the line location that best represents their experience of the phenomena. The distance of this mark from the end-point(s) is measured to obtain a numerical value on the scale representative of the participant’s subjective experience. The goal is to obtain measures on an interval scale [2]. Examples of VAS formats, including graphic rating scale, are included in Appendix 1.

VAS has been applied in many different disciplines with origins in the field of psychophysics, where this form of scaling has been used to measure responses to sensory stimulation, such as light, sound and heat [3, 4]. Hayes and Patterson are often identified as the first to use the VAS in measurement [5]. It has further expanded to the psychometric field to measure subjective phenomenon such as feeling and attitudes. In the 1960s, VAS became widespread for measures of health-related domains such as pain, mood, depression, anxiety and alertness [3, 4]. By the 1970s, the VAS has been adopted as a tool to provide values for health states and health status measures [3]—various VAS formats were used for preference elicitation during this era [6, 7].

EQ VAS, as part of a widely used health status measure EQ-5D, is a VAS of self-assessed general health: it asks respondents to rate their health today on a vertical thermometer from 0 (worst health you can imagine) to 100 (best health you can imagine) [8]. EQ VAS is a well-established method for measuring and comparing health amongst the general population, participants from clinical trials and patients from specific disease groups [9, 10]. Furthermore, EQ VAS is regarded as a patient-reported outcome (PRO) [11] and is distinguished from most PRO measures as its numerical rating of health is reported directly by the respondent and is not subject to any external scoring/weighting system.

While empirical evidence on the EQ VAS has focused on measurement properties such as reliability, validity and responsiveness, and nearly always with a focus on the 5 dimensions of the EQ-5D profile, its conceptual foundations have not been systematically investigated. Furthermore, the EQ VAS is often used as a tool to validate and/or develop the EuroQol suite of instruments [12] but without a clear understanding of the conceptual framework for the EQ VAS itself. The objective of this paper is to report on the origin of the EQ VAS and current understanding of the EQ VAS conceptual framework via a seed paper citation literature search. The authors of this paper have expertise in health economics, empirical sociology, psychometrics and self-reported health measures. Thus, we adopt these disciplinary perspectives in the framing of this report with the focus on important concepts on what the EQ VAS is intended to capture and how it is currently understood.

## Methods

To trace the origins and investigate the conceptual framework of the EQ VAS, a literature review based on citation search which was conducted in two steps: (1) a citation search of published literature using seed papers and (2) a search of the EuroQol group’s grey literature. Due to the specialty of the investigated topic the study did not opt for a broader systematic review of the literature.

The citation search was conducted using four relevant seed papers that were selected based on the study team’s experience in the field as well as relevance of the publications [4, 13–15]. All published works that cited these seed papers up to October 2021 (forward selection) and from the seed papers’ reference lists (backward selection) were identified and their abstracts retrieved. After removal of duplicate reports, two researchers (YSF and LHC) independently screened all identified publications using title and abstract, followed by full text screening. Publications were screened using pre-specified exclusion criteria, which were: (1) did not study adults, (2) did not relate to the EQ VAS, and (3) did not address the measurement framework(s) for the EQ VAS. An a-priori extraction table was used by the two reviewers to record information from the included full-text publications. Information on study design, patient characteristics, and study results, was extracted.

In parallel to the citation search of published literature, a grey literature search of EuroQol conference proceedings was conducted. One of the challenges of identifying the scientific literature on concept of the EQ VAS is that much of the original scientific investigations during the early development phases of the EQ-5D instrument were not published. Although these early works were shared and discussed during EuroQol conference proceedings, these early proceedings were less formal than current meetings and navigating the grey literature depended on communications with researchers involved in early EQ-5D instrument development. Thus, YSF and LHC communicated with experts (PK, DP, RB, GB & CG) via personal interviews and email communications, as well as reviewing EuroQolus (an early EuroQol effort implemented by Erik Nord meeting notes 1987–1996, [16]) to identify relevant study reports in the period 1987–1996.

Due to the nature of the data collected, the results are presented in a narrative format to address the development of the EQ VAS, empirical evidence of a conceptual framework, and the EQ VAS designs.

### The origin of the EQ VAS

From the targeted search, a total of 27 papers were selected to inform this report [1, 4, 13–15, 17–38]. The search and selection flowchart and the summary table of the data extraction are detailed in Appendix 2 and 3. Much of the information on the origin of the EQ VAS were found from the grey literature search as well as EuroQol published materials (books).

As already introduced, VAS were already popular tools for eliciting values for health states (valuation) from individuals since the 1970’s [6, 7, 39]. From 1987 onwards, a group of researchers, later known as the EuroQol group, held regular meetings to develop a generic instrument to describe and value health, which led to the later establishment of the EQ-5D. During the early phases of EQ-5D development in 1987–90, a descriptive system to classify health was created (the current version includes five dimensions) and VAS was proposed as the preferred valuation method (other valuation methods were still in their infancy, such as time trade-off) [40]. In a book detailing the early meetings of the (EuroQol) research group, Brooks noted that the reason for choosing VAS over other valuation methods “was its relatively simplicity for scaling and quick way of getting valuations” [41]. Devlin and Parkin et al. recounted the reason for adopting VAS as the standard method: “self-completion questionnaires were seen as the only practical means of obtaining large population level valuation data sets and the VAS was the most suited to such a survey instrument” [42].

**Fig. 1:**
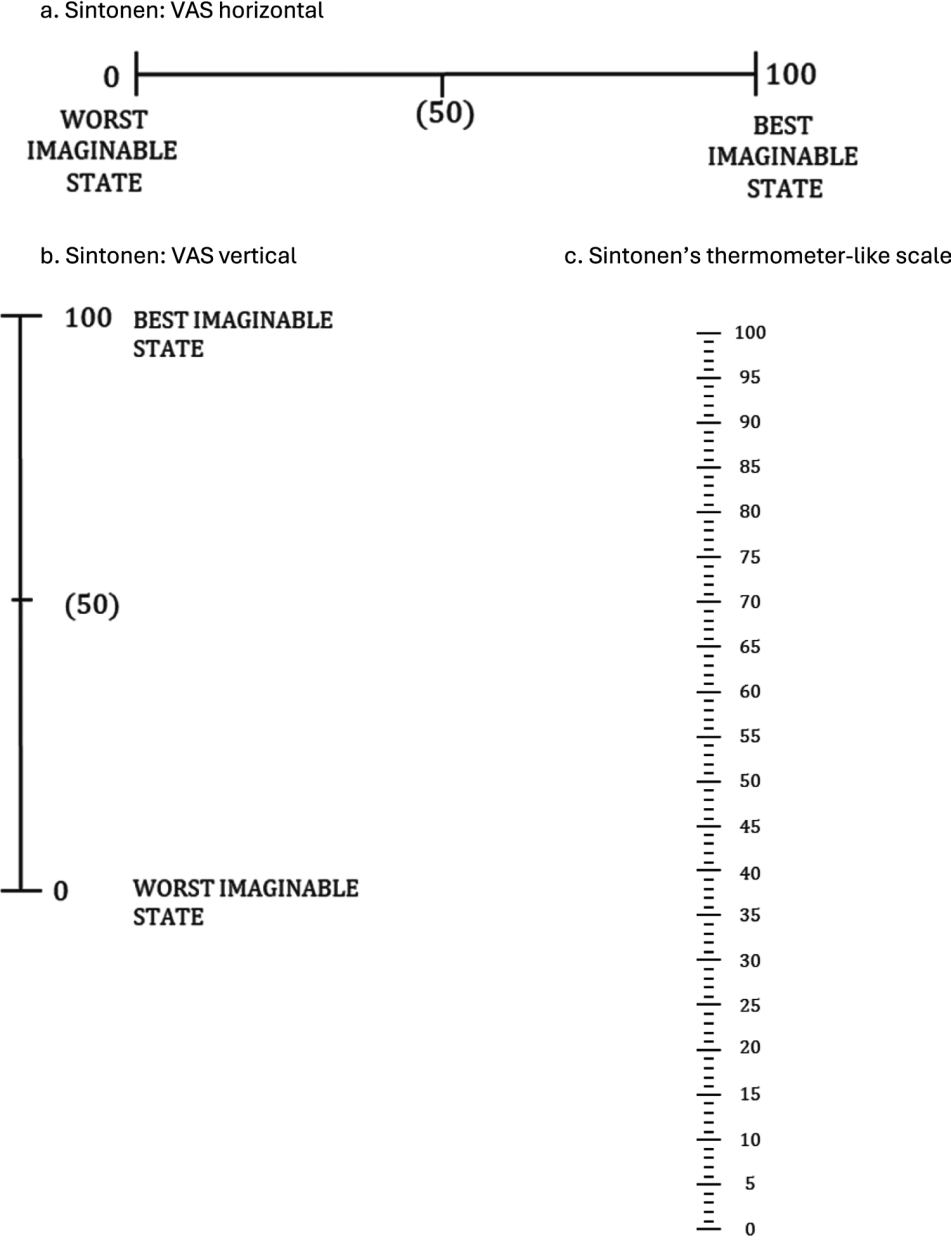
Sintonen experiment (43)

**Fig. 2:**
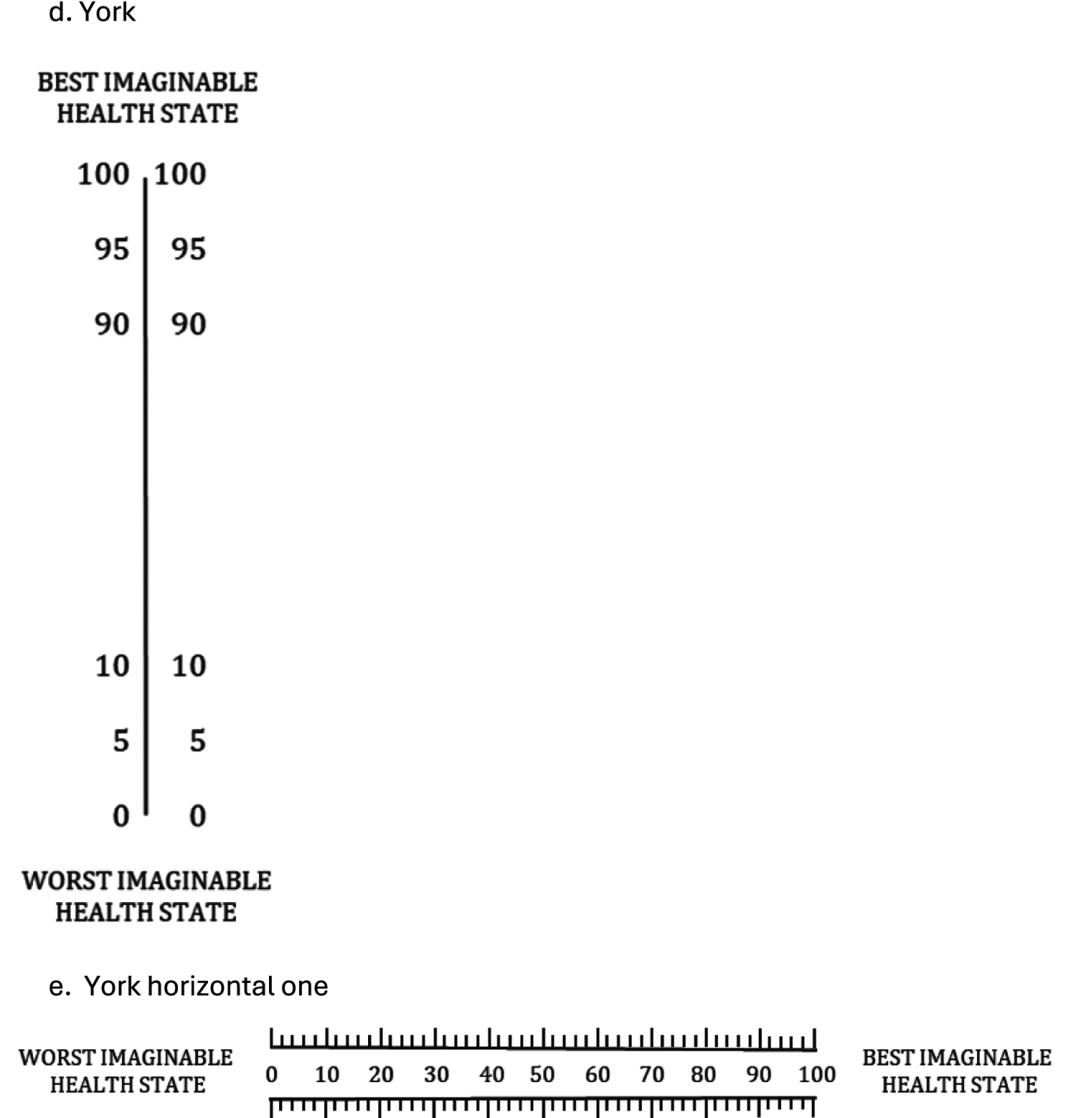
York experiment (44)

**Fig. 3:**
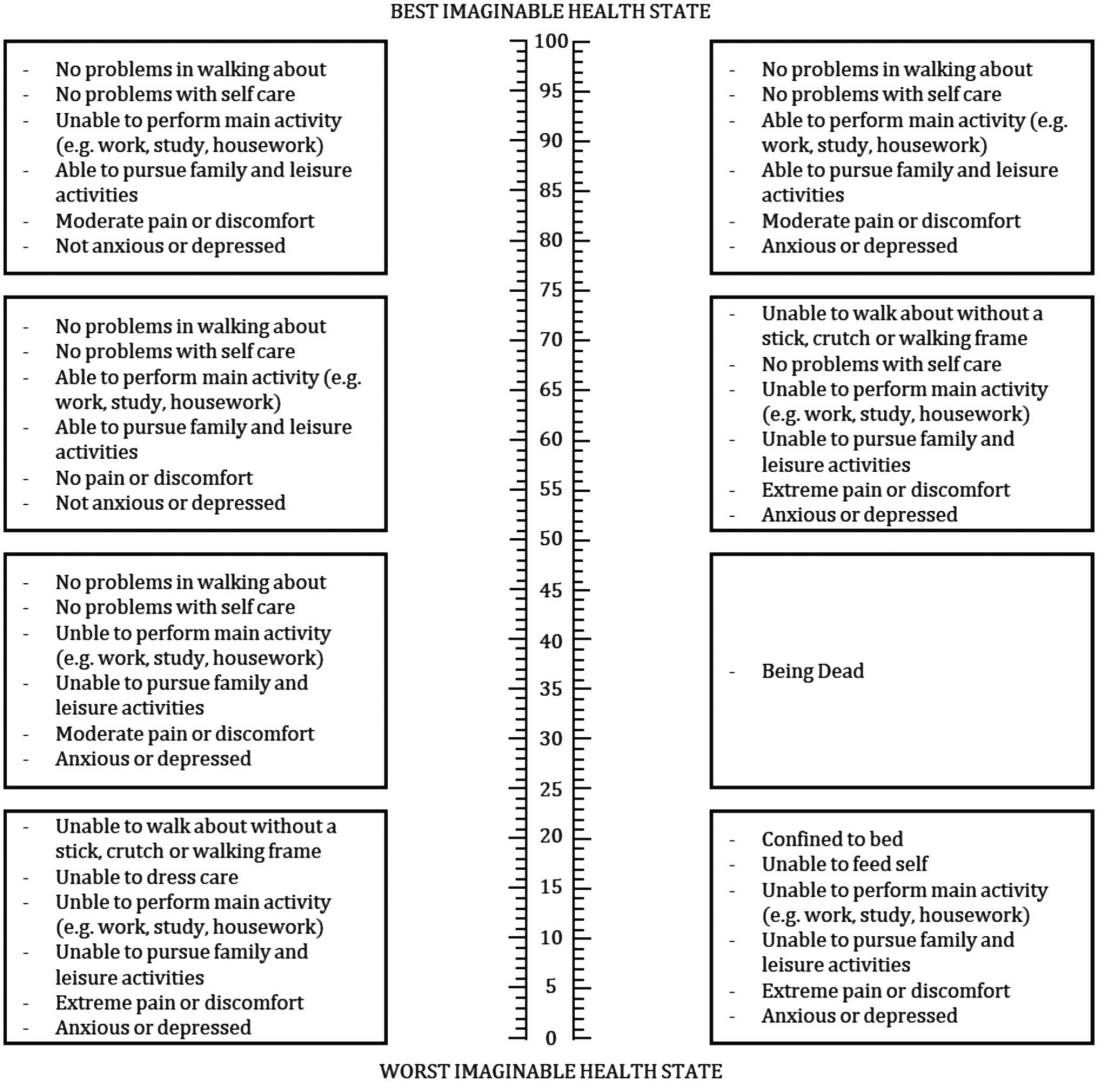
EuroQol VAS (41)

Grey literature (meeting notes) and publications documented the evaluation of a physical format of a VAS suitable for valuation. Various empirical studies tested VAS presentations. For instance, Sintonen tested vertical vs horizontal VAS, and vertical VAS with numerical labels and hash marks (much like a thermometer) were preferred (as illustrated by Fig. 1a–c) [43]. In parallel, a research team at the University of York tested the length and orientation of VAS and no difference across formats was found (as illustrated by Fig. 2d, e) [44]. Many research groups, including those in Rotterdam, Brunel and London, contributed to testing VAS design to find the most suitable format(s). By 1990 the final format of the VAS was fixed and, until current day, has not substantively changed: a single 20 cm vertical line with numerical indicators at 10-point intervals and hash marks at each millimeter with the top (100) labeled as “best imaginable health” and bottom (0) labeled as “worst imaginable health” [41], commonly known as a (feeling) thermometer or EuroQol VAS. As the valuation task in postal surveys, the EuroQol VAS was arranged in the middle of a letter-sized page with descriptions of hypothetical health states arranged on either side (reproduced in Fig. 3). Respondents were asked to draw a line from each health state to a position on the EuroOol VAS to indicate its numeric score.

**Fig. 4:**
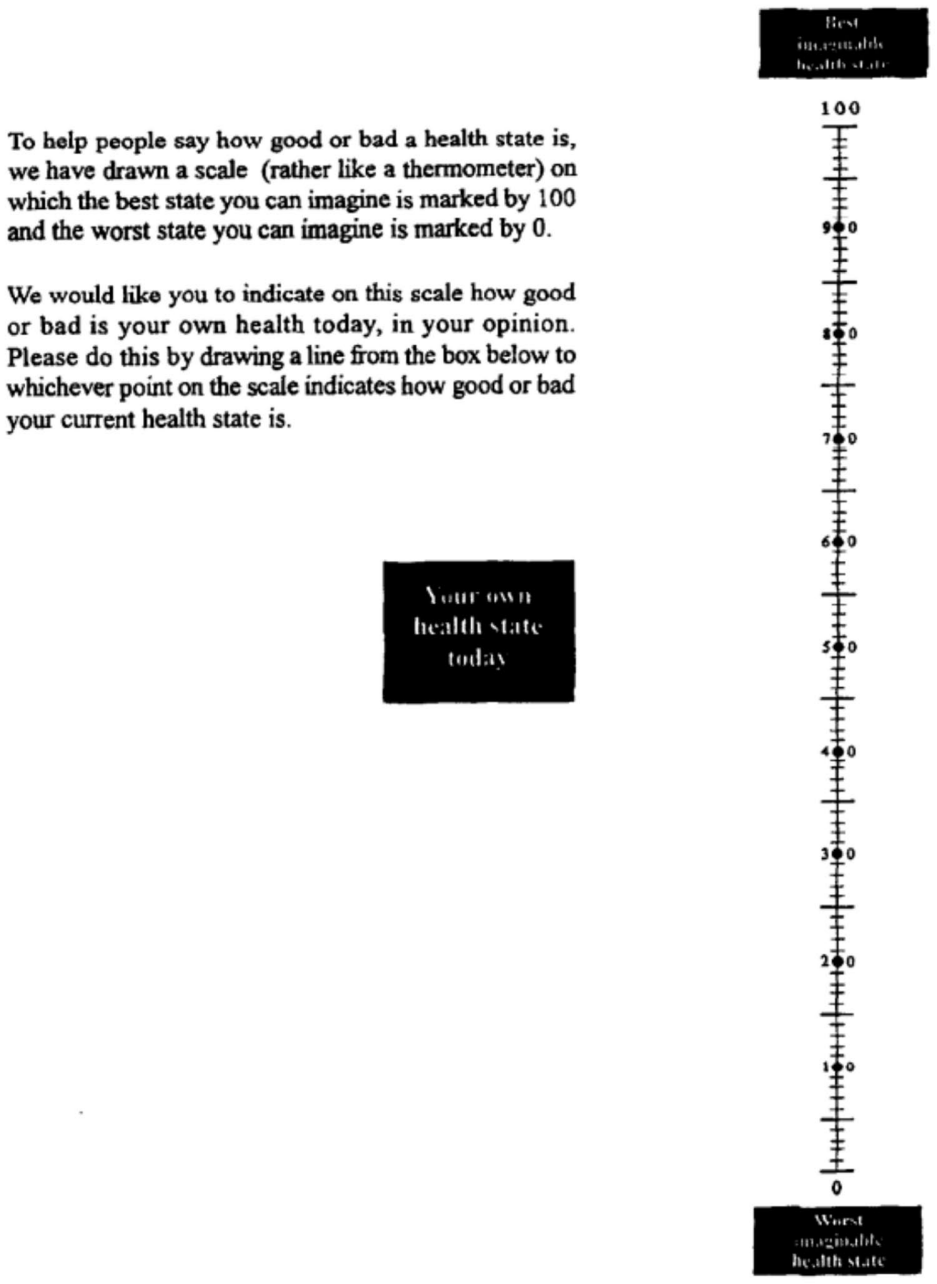
EQ VAS, 1990–2009 (41)

Prior to 1994, the EuroQol instrument comprised of the five items of the EQ-5D profile (page 2), own health rating using the EuroQol VAS (page 3), followed by the valuation exercise using EuroQol VAS to elicit values for hypothetical health states. Pages 2 and 3 served as a “warm-up” task to familiarize respondents the EuroQol health states and the EuroQol VAS tool to facilitate the valuation task. The own health item (page 3) asked respondents to draw a line from a response box to the EuroQol VAS line to rate their own general health that day (shown in Fig. 4). The box-enclosed text matched the format used for the valuation task. In early applications of the instrument, pages 2 and 3 were sometimes used “off label” as a stand-alone instrument to collect health status information in clinical studies and trials. In 1994, pages 2 and 3 were endorsed by the EuroQol group as an independent questionnaire (the EQ-5D), and page 3 became known as the “EQ VAS” [41].

**Fig. 5:**
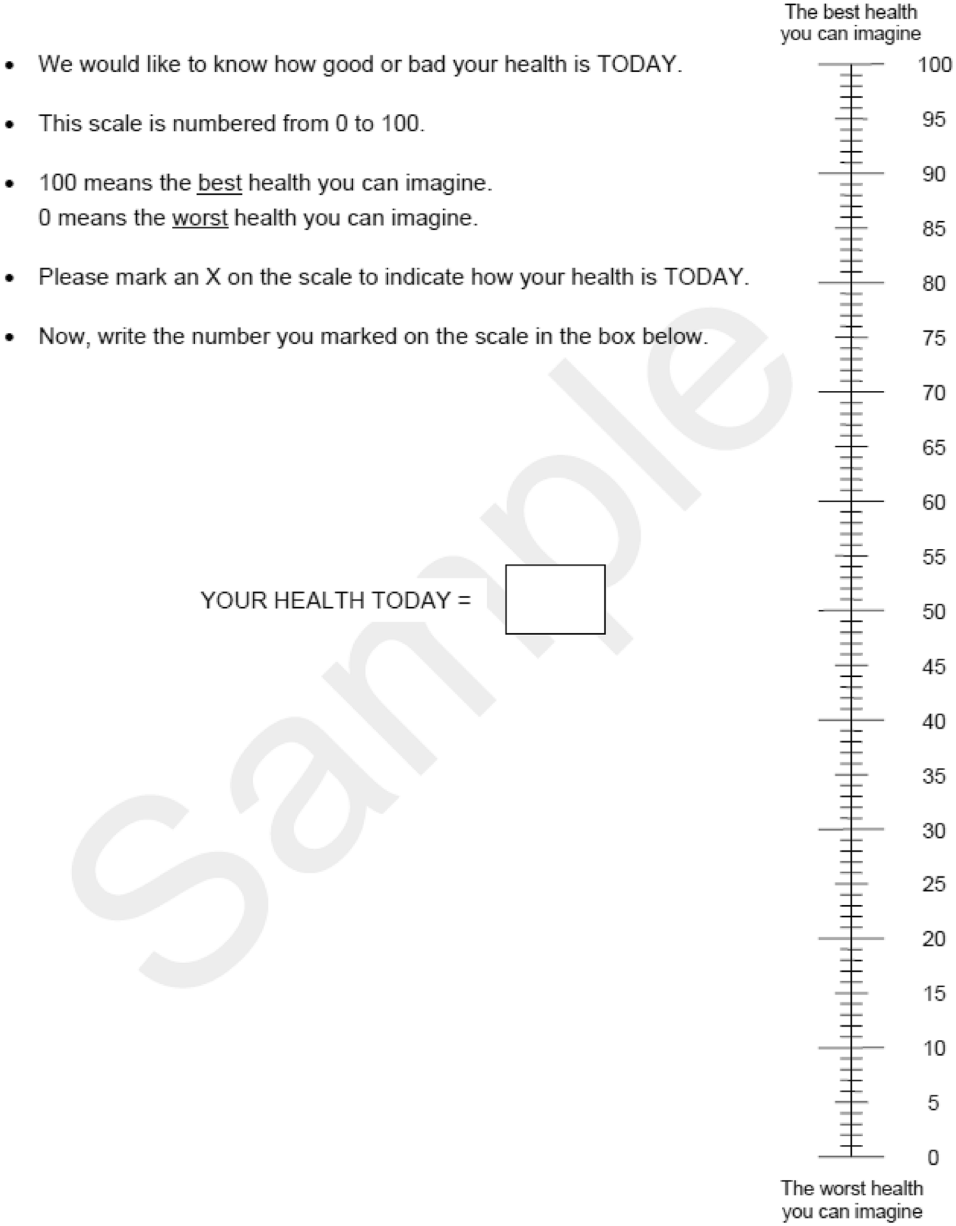
EQ VSA, current (45)

After nearly 2 decades of using EQ VAS as a self-reported health measure, a major revision occurred in 2009 due to several reporting issues. The modifications included asking the respondent to write a number into the box directly instead of drawing a line, as well as improving the text instruction. See Fig. 5 for an example of the current version of EQ VAS [45]. For a detailed report on the development of EQ-5D instrument, please refer to Brooks’s 2013 book “The EuroQol Group after 25 years” [41].

### The conceptual framework of the EQ VAS

The search for conceptual framework was mainly based on the citation search (see Appendix 2 and 3 for an overview of included literature). We found a dearth of literature addressing the conceptual framework of EQ VAS as a measure of self-rated general health. A set of included studies (n = 6) addressed the conceptual framework and measurement theories of general VAS scales not specific to the EQ VAS [1, 26–30]. One study by Karimi and colleagues established a 4-stage framework of how individuals value health states using EQ-5D, but the paper did not include the EQ VAS [19].

Two qualitative studies offer insight into what the EQ VAS measures [13, 14]. Tan and colleagues examined the cultural appropriateness of EQ VAS in three Asian countries (China, Japan and Singapore) [13], using open-ended questions (e.g. What does “best imaginable health” mean to you?) to understand participants’ interpretation of the EQ VAS. Based on 144 face-to-face interviews, they found that interpretations of “the best imaginable health” varied amongst participants and can be categorized into 5 themes: physical health, mental well-being, social relationships, medical conditions and treatment, and health promotion knowledge and behaviors. Some participants considered the “best imaginable health” to be unachievable. Interpretations of “the worse imaginable health” also varied and can be categorized into three themes: “death,” “disease,” and “disability.” In another qualitative study, Ernstsson and colleagues investigated how patients with type-1 diabetes think and reason when reporting and valuing their own current health, using EQ-5D-5L, EQ VAS, and an open-ended TTO question [14]. The face-to face interviews also found that respondents had difficulties defining, imagining, and/or relating to the “best imaginable health” label of EQ VAS. Most respondents used the best imaginable health as a reference point and related/ compared their health to it.

Amongst the empirical studies focused on EQ VAS, the majority (n = 7) investigated the difference in or associations with social-demographic, economic, behavior, health condition or environmental variables. Age, education, income, employment, drinking, and exercise were common factors which demonstrated their impact on EQ VAS scores.

Amongst studies of general VAS, one publication [30] noted that VAS, as a global scale, cannot be explained by additive measures, which echoes the more recently proposed emergent model of single-item self-rated health measures [46]. Another argue that earlier development and uses of VAS scales were intended to evaluate within-subject change but more current applications assess between-subject differences [28]. One paper [26] expressed concerns using VAS scales for utility estimations.

Many of the papers excluded during screening addressed measurement properties of the EQ VAS including construct and/or known-group validity in various health conditions. Although not directly relevant to the framework of the EQ VAS, it is important to note that these studies demonstrated satisfactory to strong validity. As found by a systematic review of the psychometric properties of the EQ VAS by Cheng et al.—using the COSMIN framework to assess the validity, test–retest and responsiveness—overall construct validity has been demonstrated across disease groups but less consistently in Asian populations [15]. The authors of the review hypothesized that this finding could be due to the greater variability in the interpretation of the EQ VAS amongst Asian respondents as observed by Tan and colleagues [13]. Rest-retest and responsiveness of EQ VAS were two areas with less satisfactory performance [15].

### The EQ VAS design

Many general VAS publications cautioned about end-point descriptors, with the earliest published paper we identified advising not to use extreme language [1]. The end-point labels of the EQ VAS with the top defined as “the best imaginable health” and the bottom as “the worst imaginable health” have been used since the EQ VAS was used as a warm-up task for valuation. Qualitative studies found that these labels are difficult to imagine or relate to, or even be considered as unachievable (for the best imaginable health) [13, 14], leading to avoidance of the end-points of the EQ VAS. Likely this issue may explain the phenomena that among respondents reporting no problems on the EQ-5D descriptive system, the average EQ VAS scores are further away from its full score of 100 [17, 47]. This finding has often been interpreted as the EQ VAS covering more aspects of health than the 5-dimensions of the description system [48], but could also be due to end-point aversion bias. Furthermore, “the best imaginable health” and “the worst imaginable health” capture two different concepts and applying both to a single continuum is a bipolar design. Wewers and Lowe [28] cautioned that bipolar designs are less effective than unipolar designs, where only one concept is applied to the continuum (e.g. “no pain” and “extreme pain”). Ernstsson and colleagues found some respondents to be less attentive to the “worse imaginable health” endpoint than the “best imaginable health” endpoint [14]. The use and impact of end-point label of the EQ VAS warrant further empirical research.

EQ VAS includes hash marks (scale marks) to indicate integers from 0 to 100 with larger marks at deciles. One of its earliest “thermometer-like scale” applications for valuations was the study by Torrance (1976) where a category scaling method—a 100 mm “desirability line”, assuming 101 equal-interval categories (also known as equal-interval scaling)—was used [6]. A similar form of the design was borrowed by EuroQol group. In comparison to a traditional or general VAS (a straight line without hash marks or numbers, Fig. 1a and b), researchers can easily ascertain the numerical value indicated by the respondent without the need of a ruler to measure the location of the mark on the scale line. However, Freyd in a 1923 publication advised against using breaks and division lines in rating scales [1]. It is interesting to note that Tan et al.’s qualitative study found that some respondents reported the hash marks of the EQ VAS to be “too granular”: they recommended that removing hash marks may make the scale easier to understand [13]. However, it reminds unexplored whether this finding can be generalized. The impact of EQ VAS scale marks and whether they lead to an optimal response scale design is in need of further research.

## Discussion

Over the past 30 years, new forms of EQ-5D and its modes of administration have been designed and developed [49–51]. Despite these changes, self-health rating using the EQ VAS remains an ever-present component of EQ-5D instruments [41]. The analysis and interpretation of EQ VAS has also been published [52]. Data collection over the past 3 decades has created thousands of EQ VAS observations and yet, notwithstanding this accumulated information, an elusive question remains unanswered—what does EQ VAS measure? Feng et al. reflect on this same fundamental question (see p. 970 in [4]) but do not provide a complete answer. Instead, they put forth the reasonable theory that the EQ VAS captures dimensions of health beyond the EQ-5D 5-dimension classifier.

This paper aims to describe the origin of the EQ VAS and to understand its conceptual framework. Our findings indicate that the self-reported EQ VAS was originally used as a warm-up task for valuing hypothetical health states. The characteristics of the EQ VAS reflect its valuation origin, such as drawing a line (previous version), vertical orientation, and choice of end labels: none of these design elements were chosen for the purpose of measuring self-reported overall health. Despite this, the EQ VAS proves to be a valid self-reported health status measure with its psychometric properties demonstrated in various general and clinical populations [15, 25]. Whether the current format of the EQ VAS is optimal in measuring self-rated overall health needs further examination. As manifested in some concerns over the design of the EQ VAS, such as the end-point label and scale marks, future research focusing on these design elements is warranted. At the same time, one should be aware of the implications of changing a long-standing instrument, such as the EQ VAS. Being a consistent and standardized instrument over time has lent strength and rationale of EQ VAS in measuring self-reported health status. As one of the main uses of EQ VAS is to understand how results compare across conditions or trials, having this historic reference is widely beneficial. Thus, any potential gain from changing its current design/format should be carefully weighed against its likely losses.

Furthermore, due to particularities of online platforms, the currently available digital versions of the EQ VAS can have large differences from the paper versions. For instance, some of the digital versions require the respondent to use a sliding scale to indicate a position on the VAS, while other versions have fewer or nearly no hash marks and/or the accompanying numerical labels along the length of the scale. It is uncertain whether/how these differences impact measurement and the equivalence of each online version with the paper format. The EuroQol group is currently undertaking the task of harmonizing the digital EQ VAS versions. Meanwhile, evidence that modern electronic survey platform may be beneficial for VAS has developed outside of self-rated health measures, some demonstrating that VAS is an interval scale with superior measurement properties than Likert-type response scales [53–55]. However, specific investigation on digital EQ VAS formats is needed.

There is a dearth of published literature addressing the conceptual framework of EQ VAS as a measure of self-rated overall health. Its similarity to the single measure of self-rated health (SRH) using an ordinal response format, alongside its widespread use due to being included in the EQ-5D suite of instruments, make it an important measure to better understand and utilize [56]. For the past half century, SRH has been shown, across a large variety of populations, to be one of the strongest predictors of mortality and morbidity [57–59] even after accounting for a myriad of other risk factors. In addition to empirical research, much theoretical framework regarding what this SRH item is measuring and why it has such a strong relationship with morbidity and mortality has been developed including cognitive, social, and emergent models [46, 56, 60–62].

The single-item SRH is similar to the EQ VAS in concept as both items ask respondents to provide an overall assessment of their own health. It is possible that much of the theoretical framework of SRH also applies to the EQ VAS. If, conceptually, the EQ VAS captures a similar phenomenon as the single-item SRH, then it potentially can be as powerful measure of health. Only one paper exploring if EQ VAS can predict mortality has been found [62]. More research on the applicable of the theoretical framework of SRH on the EQ VAS can further our understanding of the EQ VAS.

## Conclusion

EQ VAS is a valid measure for self-assessed general health status despite its origins, designed as a warm-up task for valuing hypothetical health states. Whether its current format is optimal for measuring self-reported overall health is under-investigated and not well understood. With its potential as a powerful measure of overall health, further research into its design, conceptual framework and empirical function is warranted.

## Supplementary Information

Below is the link to the electronic supplementary material.

Supplementary file1 (DOCX 233 kb)

## Data Availability

Not applicable.
