# Supplementary material for: Exploring the origin and conceptual framework of the EQ VAS
Source: Qual Life Res. 2025 Apr 26;34(8):2163–73. doi: 10.1007/s11136-025-03947-6 (PMC12274267; doi:10.1007/s11136-025-03947-6)
Supplement: Supplementary file 1 — Supplementary file1 (DOCX 233 kb) [file 11136_2025_3947_MOESM1_ESM.docx]

Appendix 1.

1. VAS (horizontal)


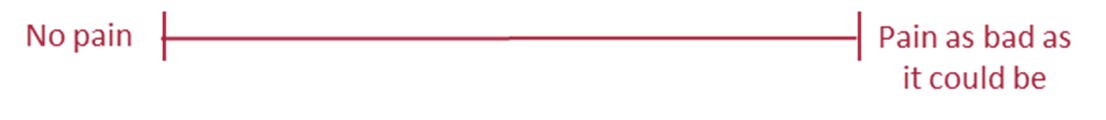


1. Graphic visual scale


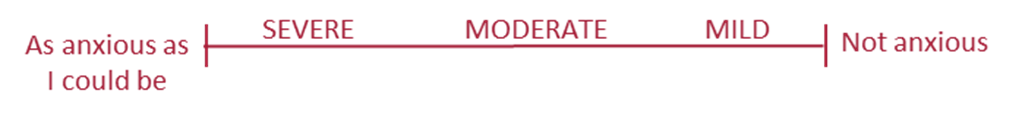


1. VAS (vertical) d. Numerical rating scale


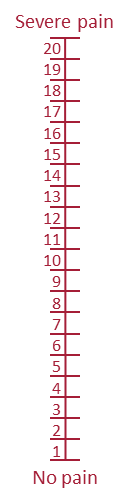

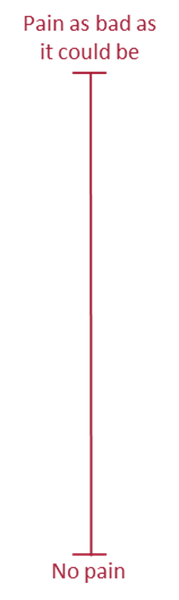


As shown in Appendix 1 a and b, taken from Wewers and Lowe [28], in the case of measuring “pain”, VAS is a horizontal or vertical line with two extreme labels “No pain” and “Pain as bad as it could be”. Graphic rating scale” is shown as figure 1c, which presents as a VAS line with several text descriptors across the length of the line. Some name another type of VAS, where numerical values and sometimes hash marks are added to the line as indicators of distance, as “numerical rating scale”, as shown in figure 1d [28].

Appendix 2. Data extraction

| **Author, year** | **Country** | **EQ VAS or others** | **Theoretical or empirical** | **Qualitative or Quantitative** | **Sample Size** | **Sample type** | **Summary of Findings** |
| --- | --- | --- | --- | --- | --- | --- | --- |
| Feng 2014 [4] | UK | EQ VAS | Empirical | Quantitative | 331,951 | Patients in England undergoing four elective surgical procedures, both before and after surgery. | Summarizes EQ VAS origin. Label and reporting pattern (response type) of VAS was analyzed.  Recommendation on improving VAS data quality EQ-5D dimensions account for 27% of variance of EQ VAS. EQ VAS is a better overall measure of patient's health than profile. |
| Cheng 2021 [15] | Review: Global studies | EQ VAS | Empirical/  Review | Quantitative | 50 papers | Publications | Corelates of VAS and quality of evidence differed across countries. However, overall, construct validity was demonstrated across disease groups but less consistently in Asian populations. This could be due to the interpretation of EQ VAS. |
| Tan 2021 [13] | East Asia: China, Japan, Singapore | EQ VAS | Empirical | Qualitative | Chinese n=69; Japanese n=24; Singapore n=20 | General public | Domains used to determine the meaning of 100 in EQ VAS: physical, mental health, behaviors, treatment. Japanese participants did NOT use Social relationships for 100. Reasons for not choosing 100 were fatigue, stress, discomfort, having a medical condition. Meaning of 0: interpreted as death/ near death/ very severe disability. Positive feedback: EQ VAS easy to understand, good tool for people to reflect on their health. Negative Feedback: scale is too granular; number points open to interpretation. |
| Ernstsson 2021 [14] | Sweden | EQ VAS | Empirical | Quantitative | 20 | Patient with type 1 diabetes | Respondents had difficulties of defining/imagining/relating to the “best imaginable health” label of EQ VAS. Most respondents used the best imaginable health as a reference point and related/ compared their health to it. Less attention was giving to the worse imaginable health. |
| Aitken 1969 [30] | UK | OTHER VAS | Theoretical | NA | NA | NA | Health measurement in general is "the assignment of numerals to things so as to represent facts and conventions (Stevens 1946). Discrete scores are not additive so the VAS, a global scale, cannot be explained by additive measures. VAS is sensitive to clinical interventions and useful, especially as it can be more precise than other measures. |
| Freyd 1923 [1] | NA | OTHER VAS (Psychological experiments) | Theoretical | NA | NA | NA | Describes older study of mental imagery and its relationship with descriptive response choices in rating scales. Both statistical and nonstatistical (ease of use, popularity, etc). A merit of rating scales is the absence of quantitative terms. Of many suggestions for such a scale, those pertinent to the EQ VAS are: there should be no breaks or divisions on the line; descriptive endpoints should not be extremely worded (as never to be employed). Cautions on bias such as framing and "halo" |
| McCormack 1988 [29] | Australia | OTHER VAS | Empirical/  Review | NA | NA | NA | The history of VAS is well illustrated in this review. Started by Hayes & Patterson 1921 and Freyd 1923 but becoming widely use after promoted by Aitken & Zealley 1969. Demonstrating the validity of VAS, in terms correlation (with other measures), discriminative capacity and reliability. Interesting, the author questioned the scaling of VAS scale - it seems that the 100 scaling recommended by Aitken is just a convenience choice and called for more studies. It also discussed the scale property of VAS - ordinal, interval or ratio scale. |
| Wewers and Low 1990 [28] | USA | OTHER VAS | Empirical/  Review | NA | NA | NA | Reporting history and psychometric property, statistics (scaling, parametric vs nonparametric, normalization) and discuss limitations. VAS was originally designed to measure within-subject change; later was used for between-subject change. most of earlier studies applied in pain, mood, anxiety and depression researches. |
| Torrance 2001 [26] | Canada | OTHER VAS | Editorial/  Review | NA | NA | NA | Sciences/economics theoretical foundation (Robinson et al. 2001); psychology and psychophysics foundation (von Winterfeldt and Edwards 1986). The author did not believe VAS has a cardinal value, nor VAS can be an independent measure for Health Economics. The paper discussed 3 major problems of VAS: 1. End label problem, causing compatibility issue, 2. bias: context bias (can be corrected) and the end aversion bias, 3. not the same as SG (can be converted). |
| Robinson 1997 [18] | UK | EQ VAS | Empirical | Mainly qualitative | 45 | Participants from the MVH study | The study tried to answer three questions emerged from the MVH study and two related to VAS: 1. Why are some states rated better than dead on the VAS often rated as worse than dead in TTO? 2. Why are some respondents unwilling to trade off any time at all in order to avoid a health state that they place below 11111 on VAS?  Their findings are mostly around TTO. But one is interesting about why VAS show difference (ordinal) results than TTO is because often the preference is "constructed" rather than "reveal" - the way health states are presented in VAS (many at once), participants filled/edited out some information of health states... |
| de Boer 2004 [27] | Netherlands | OTHER VAS (horizontal) | Empirical | Quantitative | 83 | Patients with esophageal adenocarcinoma undergoing | Comparing the psychometric property of a horizontal VAS with Medical Outcomes Study Short Form-20 (MOS SF-20) and Rotterdam Symptom Check-List (RSCL). Offer a good history/background of VAS but a bit different from previous review |
| Devlin 2004 [20] | New Zealand | EQ VAS | Empirical | Quantitative | 1360 | General public | Topics covered the valuation task but not EQ VAS itself. It was concluded that the valuation exercise imposes a substantial cognitive burden on respondents and many do not understand it (by post using VAS) |
| Whynes 2008 [17] | UK | EQ VAS | Empirical | Quantitative | about 3000 | Women with low-grade abnormalities detected on screening for cervical pre-cancer (from the TOMBOLA trial) | VAS score as the dependent variable. Independent variables comprised EQ-5D health state classifications, distress, locus of control, and socio-demographic characteristics classified the extra factor. Provided some review on how other factors (age, education, race, etc.) influence VAS scores.  The paper also investigated what influence the change of VAS scores |
| Papaioannou 2011 [21] | Review | EQ VAS | Empirical/  Review | Quantitative | 33 Papers | Mixed | Some evidence that the responsiveness and distribution properties of the EQ VAS are better than the EQ-5D index |
| Luo 2012 [24] | Singapore | EQ VAS | Empirical | Quantitative | 335 Chinese- and 298 English-speaking | Diabetes patients in primary care | EQ VAS performed differently for Chinese-speaking and English-speaking Singaporeans (less sensitivity) in terms of know-group validity.  Authors speculate that Chinese speakers may avoid the end-points |
| Tran 2012 [23] | Hanoi, Vietnam | EQ VAS | Empirical | Quantitative | 1016 | Patients with HIV/AIDS | 5L VAS is valid and associated with a number of sociodemographic characteristics: female sex, lower education attainment, and unemployment. |
| Whynes 2013 [32] | UK | EQ VAS | Empirical | Quantitative | 3851 | Patients with stroke, low back pain colposcopic investigation, cytological surveillance | Regressed VAS into 5 dimensions of profile, with the inclusion of intercept and slope dummy variables specific to conditions; the results show coefficients differ according to the conditions. Implication is the same state to state change is valued different by pts' experience such as VAS. |
| McCaffery 2016 [36] | South Australia (South Australia Omnibus Survey) | EQ VAS | Empirical | Quantitative | 2908 | General population of south Australia | Distribution very close to the Health Survey of England. 5L picked up differences in gender/age while EQ VAS did not. U shaped distribution with EQ VAS and age -- similar to patterns of Well-Being EQ VAS differentiated across ALL SES variables explored. Hypothesized that EQ VAS is better for population measure as only 4.7% rated "perfect health" on VAS while 47.6% reported 11111 |
| Karimi 2017 [19] | UK | NA (more about health states rather than VAS itself) | Empirical | Qualitative | 21 | From community organization, school staffs, students, etc. | Proposed a frame for health state evaluation:  (i) interpretation and concretization of a health state, (ii) conversion factors, (iii) non-health consequences of health states and the weighing of the consequences, (iv) an implicit explanatory account the six most frequently mentioned consequences (activities, enjoyment, independence, relationships, dignity, and avoiding being a burden) are closed to other measures, such as the ICECAP-A or WEMWBS domains than the EQ-5D domains. |
| Wang 2017 [22] | Yunnan, China | EQ VAS | Empirical | Quantitative | 101 | Pregnant women who were HIV positive | Even for those with no-problems on the EQ-5D profile, the VAS (mean about 50-60) could differentiate across groups with higher and lower health on the SF-12 |
| Van Dongen 2019 [33] | Patients with chronic back pain | EQ VAS | Empirical | Quantitative | 5037 | Chronic low back pain | "Disagreements" between reported EQ VAS, VAS-dead and VAS-11111 increased in patients with more comorbidities and lower function.  Patient values tend to be a bit higher than the general public. |
| Huang 2019 [37] | Taiwan | EQ VAS | Empirical | Quantitative | 1457 (randomly sampled) | Adults 65 and older | Environmental characteristics (social welfare & cultural facilities) associated with EQ VAS but not EQ-5D profiles |
| Qian 2019 [25] | Review: Asian studies | EQ VAS | Empirical | Quantitative | 79 papers, 1504 subjects | South and East Asian studies | Feasibility for the EQ VAS not optimal in Asian countries due to low educational levels validity in Asian countries is suboptimal.  "Best imaginable health" is interpreted in broadly different ways. Missing values do not seem to be a problem. |
| Zrubka 2019 [31] | Hungary | EQ VAS | Empirical | Quantitative | 194 | Hungarian general population | Acceptable Health Curve similar across age groups but not imagined years (when references are used). Differences also across poor, average and good health (largest differences) than referenced years. Modified VAS which asks respondents to indicate the level of health acceptable at various ages can be important for understand VAS responses |
| Choi 2020 [38] | Koren | EQ VAS | Empirical | Quantitative | 13437 | Adults without activity limitations | Korean survey cross-sectional data, investigating the impact of exercise on HrQol. The data show exercise has positive associated with VAS |
| Pyo 2021 [35] | Korea | EQ VAS | Empirical | Qualitative | 18 | Physicians and nurses from medical institutions | 5-dimensions of the EQ-5D not sufficient. Positive feedback for time frame of "today". |
| Teni 2021 [34] | Sweden | EQ VAS | Empirical | Quantitative | 25867 | Swedish general population | Socio-economic (education and income), health behavior factors (smoking, drinking and exercise), self‑reported diseases and conditions related to EQ VAS |
